# Predicting relative efficacy of anthracyclines and taxanes in breast cancer neoadjuvant AC-T chemotherapy using longitudinal MRI radiomic model

**DOI:** 10.3389/fonc.2025.1544833

**Published:** 2025-05-15

**Authors:** Kaiwen Liu, Ran Zheng, Jiulou Zhang, Siqi Wang, Yingying Jin, Feiyun Wu, Jue Wang, Shouju Wang, Xiaoming Zha, Yuxia Tang

**Affiliations:** ^1^ Department of Radiology, The First Affiliated Hospital with Nanjing Medical University, Nanjing, China; ^2^ Department of Breast Disease, The First Affiliated Hospital with Nanjing Medical University, Nanjing, China

**Keywords:** breast cancer, neoadjuvant chemotherapy, magnetic resonance imaging (MRI), radiomics, longitudinal analysis

## Abstract

**Background:**

Neoadjuvant chemotherapy (NAC) is a standard treatment strategy for breast cancer, with a commonly used regimen consisting of 4-cycle anthracycline and cyclophosphamide (AC) treatment followed sequentially by 4-cycle taxane (T) treatment. Variations in treatment efficacy are observed at different stages of AC-T regimen. Stratifying patients based on the efficacy variations could provide insights to prolong the cycle of AC or T treatment, potentially enhancing the overall efficacy of NAC. Therefore, this study aimed to evaluate the feasibility of developing magnetic resonance imaging (MRI) radiomic models for predicting the relative efficacy of AC versus T treatments.

**Methods:**

This retrospective study included 190 breast cancer patients, who were randomly allocated into a training set (n=133) and a test set (n=57). All patients received NAC treatment consisting of four cycles of AC followed by four cycles of T. Breast MRI examinations were conducted before NAC (pre-NAC), before the fifth cycle (mid-NAC), and before surgery (post-NAC). Relative efficacy was defined by comparing tumor volume change rates between the AC and T treatment stages. Radiomic features were extracted from dynamic contrast-enhanced (DCE) and apparent diffusion coefficient (ADC) images based on the intratumoral and peritumoral regions at the pre-NAC and mid-NAC stages. Radiomic models were first developed, and hybrid models were then established by integrating radiomic and clinicopathological data to predict relative efficacy.

**Results:**

For radiomic models, the Delta model demonstrated effective discrimination of relative efficacy, achieving areas under the curve (AUCs) of 0.887 [95% confidence interval (CI): 0.816-0.930] in the training set and 0.757 (95% CI: 0.683-0.817) in the test set. For hybrid models, the Delta+clinicopath model showed improved performance, with AUCs of 0.887 (95% CI: 0.873-0.892) in the training set and 0.772 (95% CI: 0.744-0.786) in the test set. The Delta+clinicopath model also exhibited favorable calibration in both sets and provided a substantial clinical net benefit.

**Conclusions:**

The hybrid model is a reliable and reproducible tool for predicting the relative efficacy between AC and T treatments in breast cancer NAC. The model could help to stratify patients for personalized adjustment of NAC regimens.

## 1 Introduction

Neoadjuvant chemotherapy (NAC) has become a standard treatment for breast cancer, primarily aiming at reducing the tumor stage and enhancing the feasibility of breast-conserving surgery (1, 2). One of the most classic NAC treatment regimens is 4-cycle anthracycline and cyclophosphamide (AC) treatment followed by 4-cycle taxane (T) treatment (3–5). During the NAC treatment process, MRI can evaluate the changes in tumor volume at different stages, providing a basis for assessing the treatment efficacy and adjusting the treatment plan in a timely manner (6–9).

During the clinical treatment process, significant individual variability in patients’ responses to the same NAC regimen is observed. The variability in response occurs not only among different patients but also within the same patient at different treatment stages. In our previous study, we found that the change rates of tumor volume on MRI during the AC and T treatment stages can respectively reflect the treatment efficacy of AC and T (10). Some tumors shrink more rapidly during the AC treatment stage, while others shrink more rapidly during the T treatment stage. This indicates that stratifying patients based on the relative efficacy of AC and T and individually prolonging the cycle of AC or T treatment may improve the overall treatment efficacy of NAC. However, complete tumor volume change rate data is only available after the completion of NAC treatment, at which point it is no longer possible to adjust the regimen. Therefore, during the mid-term of NAC treatment, predicting the relative efficacy of AC and T can provide a basis for timely adjustment of the NAC treatment plan.

MRI-based radiomic models have been successfully used to predict the treatment outcome of breast cancer NAC. Radiomics extracts abundant quantitative information from breast MRI imaging, revealing the association between tumor imaging features and clinical outcome, which is crucial for optimizing treatment regimens (11–13). However, currently, no radiomic model has been developed to predict the relative treatment efficacy of AC and T in NAC.

To achieve this goal, in this study, we included breast cancer patients at our center who received NAC with AC followed by T (AC-T). These patients underwent MRI examinations before, during, and after NAC treatment. We used the ratio of the change in tumor volume during the AC and T treatment stages to measure the relative treatment efficacy of AC and T, and developed a radiomic model based on MRI data before and during the NAC treatment to classify patients, thereby stratifying patients who may benefit from prolonged AC treatment during the NAC treatment process.

## 2 Methods

### 2.1 Study participants

**Figure 1:**
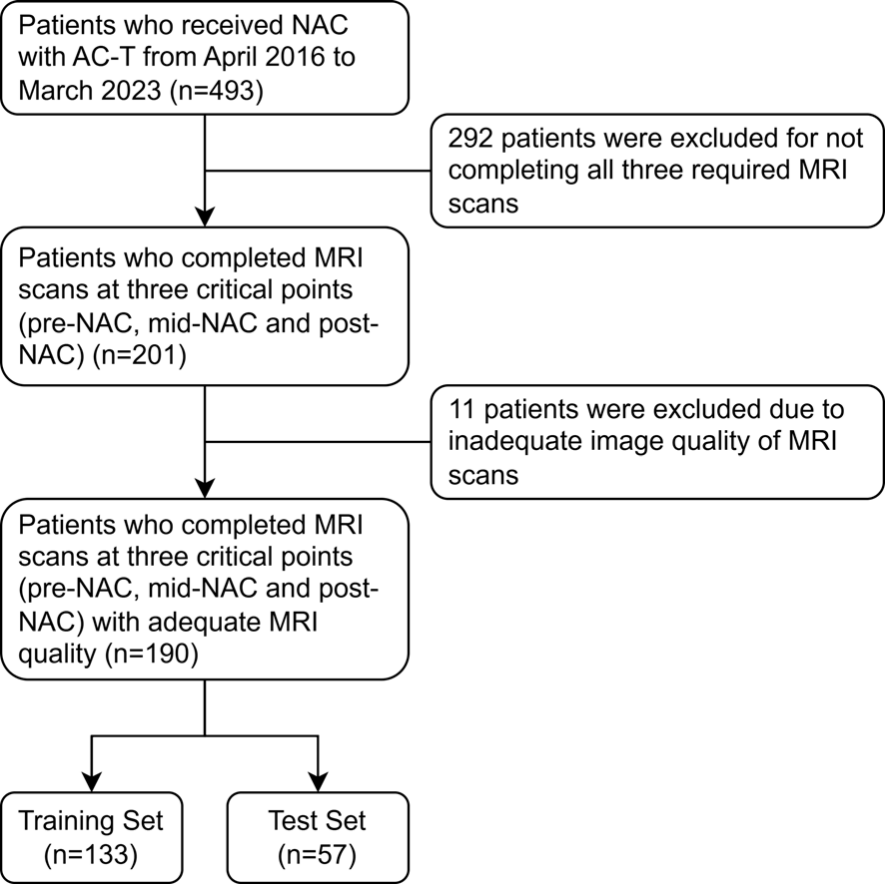
The flowchart shows the pathway for patient recruitment. NAC, neoadjuvant chemotherapy; AC, anthracycline and cyclophosphamide; T, taxanes.

This study received approval from the institutional review board of our hospital. Given its retrospective design, written informed consent was waived. **Figure 1** shows a flowchart of patient recruitment. Female patients diagnosed with breast cancer at the First Affiliated Hospital with Nanjing Medical University between April 2016 and March 2023 were included retrospectively. The eligibility criteria were as follows: operable invasive breast cancer confirmed by core needle biopsy; Estrogen receptor (ER), progesterone receptor (PR), human epidermal growth factor receptor 2 (HER2), and the nuclear protein Ki67 were evaluated by immunohistochemical (IHC) staining; Completing four cycles of AC treatment followed by four cycles of T treatment; MRI data obtained before NAC treatment (pre-NAC), before the fifth cycle (mid-NAC), as well as before surgery (post-NAC).

The exclusion criteria were as follows: inadequate MRI quality or lack of MRI data; inability to complete the full cycle of NAC due to chemotherapy-related side effects; previous chemotherapy or targeted therapy; distant metastatic lesions; unmeasurable tumor without discernible boundary; tumor scattered or discontinuous after NAC.

**Figure 2:**
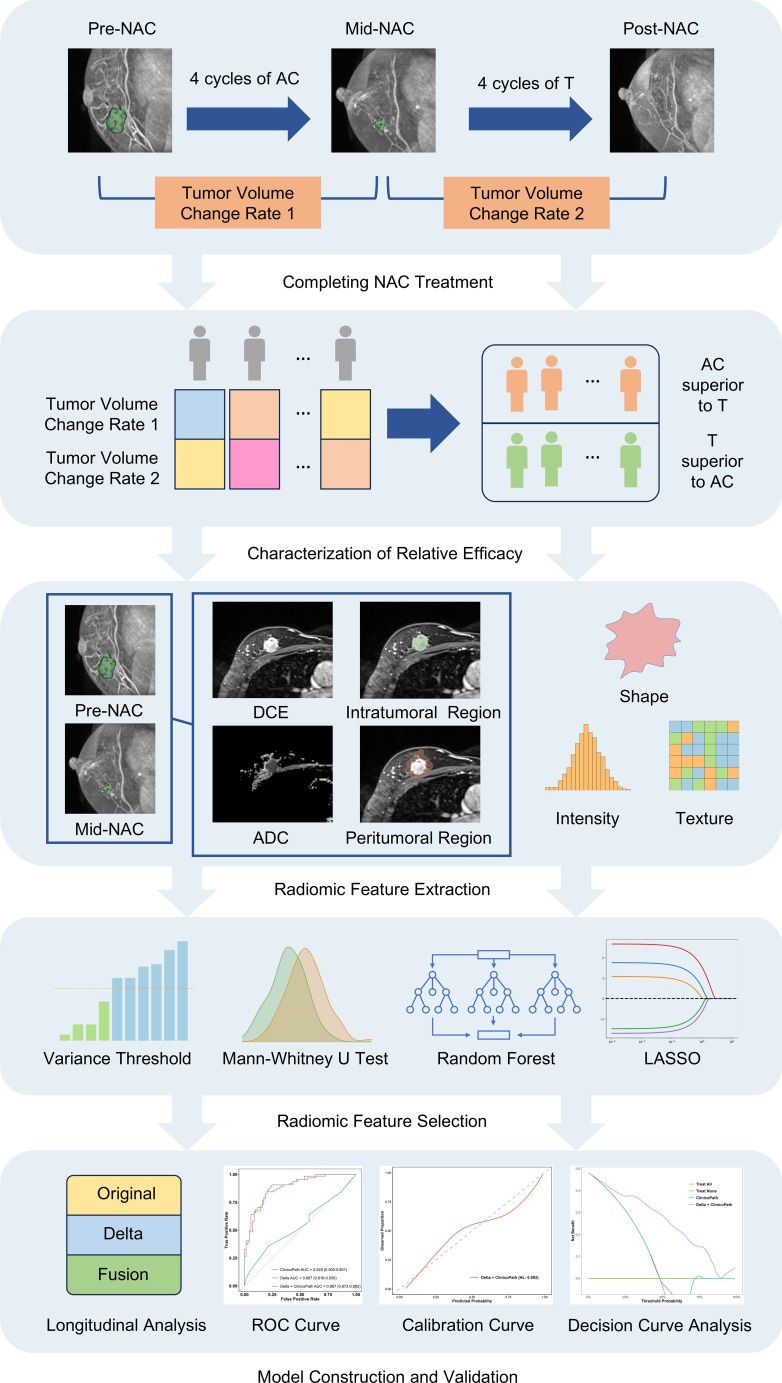
The schematic shows the study design and workflow for predicting the relative efficacy between AC and T treatments in neoadjuvant chemotherapy, using a multi-sequence, multi-region MRI radiomic model. MRI, magnetic resonance imaging; NAC, neoadjuvant chemotherapy; AC, anthracycline and cyclophosphamide; T, taxanes; DCE, dynamic contrast-enhanced; ADC, apparent diffusion coefficient; LASSO, least absolute shrinkage and selection operator; ROC, receiver operating characteristic.

Clinical data were collected for each patient, including gender, age, menstrual status, clinical T stage, and clinical N stage. Patients were randomly stratified into a training set and a test set in a 7:3 ratio. The study design and workflow are depicted in **Figure 2**.

### 2.2 Treatment strategies and pathological assessment

All patients started treatment with four cycles of anthracycline (Pharmorubicin 90 mg/m²) and cyclophosphamide (Endoxan 600 mg/m²), each administered on day 1, every 2 or 3 weeks. All patients subsequently underwent four cycles of T treatment (either nanoparticle albumin-bound paclitaxel [Abraxane 260 mg/m²], solvent-based paclitaxel [Taxol 175 mg/m²], docetaxel [Taxotere 75 mg/m²] or liposomal paclitaxel [Lipusu 175 mg/m²]), administered on day 1, every 2 or 3 weeks. HER2 positive patients received targeted therapy with trastuzumab (Herceptin 6 mg/kg with an 8 mg/kg loading dose) in addition to the T treatment.

ER, PR, HER2, and Ki67 status were determined using IHC. Tumors were classified as ER/PR-positive if they showed ≥1% nuclear-stained cells. HER2 status was assessed as negative (HER2-) with IHC grades of 0 and 1+, and positive (HER2+) with an IHC grade of 3+. For tumors with an IHC grade of 2+, HER2 gene amplification was determined by fluorescence *in situ* hybridization (FISH). Ki-67 expression was evaluated using a cutoff index of 30%; expressions below 30% were considered low, while those ≥30% were considered high.

### 2.3 MRI acquisition

MRI scans were conducted in the prone position using either a 1.5 Tesla scanner (MAGNETOM Aera XJ, Siemens) or a 3.0 Tesla scanner (MAGNETOM Trio, Siemens) at three key points: pre-NAC, mid-NAC and post-NAC. The protocol included at least diffusion weighted imaging (DWI) and fat-suppressed dynamic contrast-enhanced (DCE) sequence.

For MAGNETOM Trio, the imaging protocol included: axial DWI (repetition time [TR]/echo time [TE], 5200 ms/65 ms; matrix, 220 × 110; field of view, 323 mm × 161 mm; thickness, 5 mm) and DCE sequence (TR/TE, 4.23 ms/1.57 ms; matrix, 448 × 448; field of view, 340 mm × 340 mm; thickness, 1 mm). For MAGNETOM Aera, the imaging protocol included: axial DWI (TR/TE, 7500ms/64 ms; matrix, 180 × 84; field of view, 350 mm × 163 mm; thickness, 5 mm) and DCE sequence (TR/TE, 3.90 ms/1.66 ms; matrix, 320 × 320; field of view, 360 mm × 360 mm; thickness, 1.5 mm). Details on specific imaging parameters are available in our previous study (14).

The DCE sequence was initially acquired prior to contrast agent administration. Gadolinium-DTPA (Magnevist; Bayer Healthcare) was then administered at a dosage of 0.1 mmol/kg with an infusion rate of 3 mL/s, followed by a 20 mL saline flush. Subsequently, the DCE sequence was repeated five times. Apparent diffusion coefficient (ADC) maps were generated from DWI images using two b values.

### 2.4 Image segmentation and feature extraction

For each patient, image segmentation was performed separately on MRI images acquired at pre-NAC, mid-NAC, and post-NAC time points. Using 3D Slicer software (version 5.2.2, www.slicer.org), intratumoral regions were manually delineated slice-by-slice on the second post-contrast phase image of the DCE sequence. The intratumoral regions were then isotropically expanded by 5 mm in three dimensions to obtain the peritumoral regions using the SimpleITK package (version 2.2.1) in Python 3.9.13. A radiologist with five years of experience in breast imaging performed the segmentation for all cases. The radiologist was blinded to the clinicopathological data and the relative efficacy information. Radiomic features were extracted from both the original and filtered images using PyRadiomics (version 3.0.1, https://github.com/Radiomics/pyradiomics). Filtered images were generated using the Laplacian of Gaussian operator and wavelet filters. The Laplacian of Gaussian filter was applied with kernel sizes of 1, 2, 3, 4, and 5 for the DCE sequences and 2, 3, 4, and 5 for the ADC maps. The wavelet filter decomposed each dimension into eight levels. Radiomic features included various categories, including first-order (intensity-based histogram), shape-based, gray-level co-occurrence matrix (GLCM), gray-level size zone matrix (GLSZM), gray-level run length matrix (GLRLM), and gray-level dependence matrix (GLDM). Prior to feature extraction, the intensity distribution of images was normalized. Voxel size was resampled to achieve isotropic voxels of 1.0 mm × 1.0 mm × 1.0 mm for the DCE images and in-plane isotropic voxels of 2.0 mm × 2.0 mm for the ADC maps with the sitkBSpline interpolator. Additionally, voxel intensity values were discretized with fixed bin widths set at 5 for the DCE sequence and 25 for the ADC maps. A total of 1,218 features were extracted from each region for the second post-contrast phase image of the DCE sequence and 1,132 features for the ADC maps. Considering the two types of regions (intratumoral regions and peritumoral regions), each patient’s imaging data from pre-NAC and mid-NAC images contributed a total of 9,400 features from both the DCE and ADC images. To capture longitudinal tumor changes, delta radiomic features were calculated as the differences between the radiomic feature values from the pre-NAC and mid-NAC images. As a result, each patient yielded a total of 14,100 radiomic features.

### 2.5 Characterization of relative efficacy

**Figure 3:**
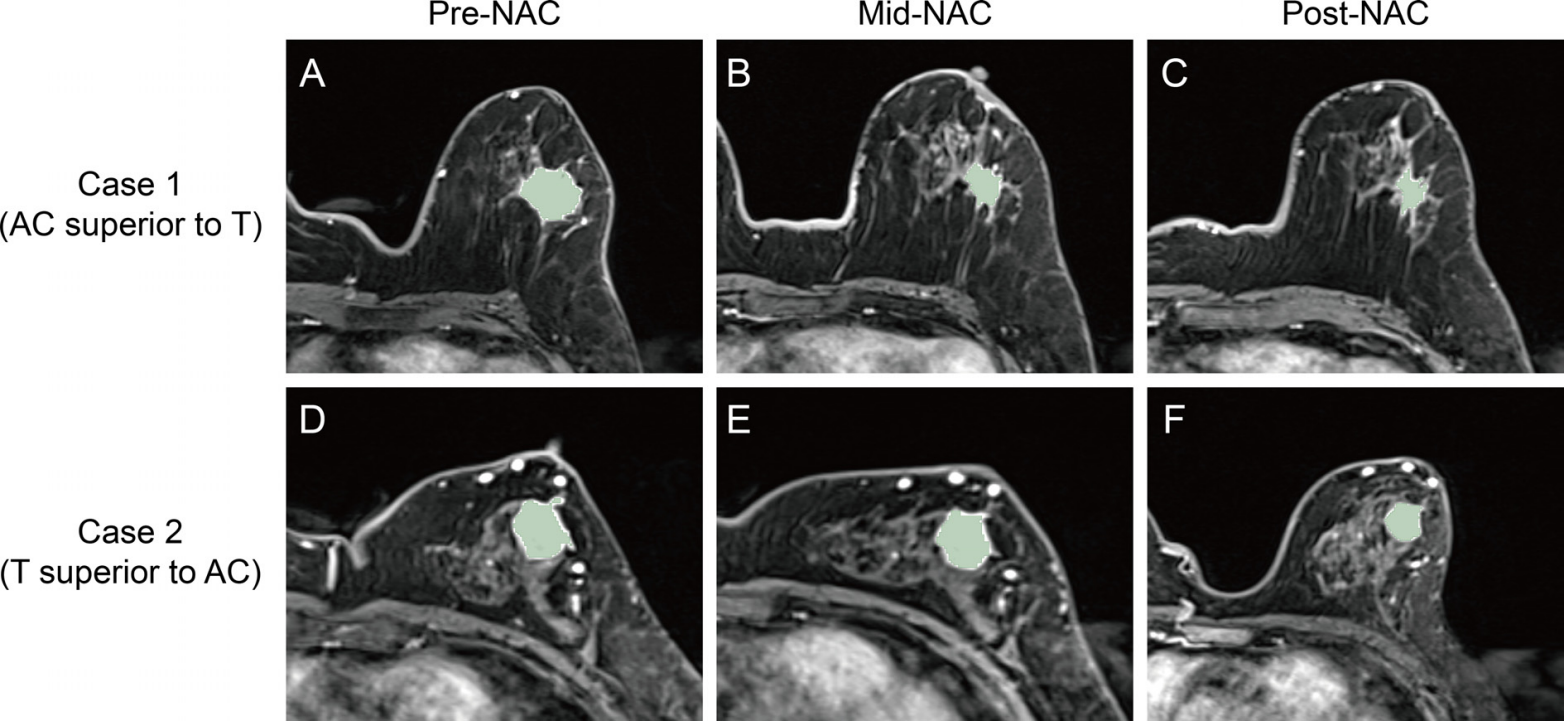
Second post-contrast phase images of the DCE sequence and corresponding intratumoral regions from the two cases exhibiting different types of relative efficacy. For Case 1, whose lesion was imaged at pre-NAC **(A)**, mid-NAC **(B)**, and post-NAC **(C)** stages, the relative efficacy was considered to be AC superior to T. For Case 2, whose lesion was imaged at pre-NAC **(D)**, mid-NAC **(E)**, and post-NAC **(F)** stages, the relative efficacy was considered to be T superior to AC. DCE, dynamic contrast-enhanced; NAC, neoadjuvant chemotherapy; AC, anthracycline and cyclophosphamide; T, taxanes.

Tumor volume was derived from Mesh Volume feature within the shape category of radiomic features. Mesh Volume feature calculates the volume of a structure by estimating the volume enclosed within a 3D mesh model. The efficacy of the four cycles of AC treatment was determined by calculating the relative net reduction in tumor volume from pre-NAC to mid-NAC (δ_AC_). Similarly, the efficacy of the four cycles of T treatment was calculated using the relative net reduction in tumor volume from mid-NAC to post-NAC (δ_T_). Relative efficacy is defined by the ratio of δ_AC_ and δ_T_. If the ratio of δ_AC_ and δ_T_ is greater than 1, the patient is considered more sensitive to AC treatment stage (AC is superior to T). Otherwise, the patients is considered more sensitive to T treatment stage (T is superior to AC). **Figure 3** and **Table 1** show examples of two cases exhibiting different types of relative efficacy.

**Table 1:** Tumor volume change data of the two cases exhibiting different types of relative efficacy.

Case	Tumor volume (mm^3^)			δ_AC_	δ_T_	δ_AC_/δ_T_	Relative efficacy
	Pre-NAC	Mid-NAC	Post-NAC				
Case 1	9071.81	2194.61	937.44	0.76	0.57	1.33	AC superior to T
Case 2	2933.88	2481.79	1666.43	0.15	0.33	0.45	T superior to AC

NAC, neoadjuvant chemotherapy; AC, anthracycline and cyclophosphamide; T, taxanes; δ_AC_, the relative net reduction in tumor volume from pre-NAC to mid-NAC; δ_T_, the relative net reduction in tumor volume from mid-NAC to post-NAC.

### 2.6 Radiomic feature selection

To ensure inter-observer consistency, a second radiologist with 5 years of experience re-segmented a randomly selected subset of 50 cases. Both radiologists were blinded to the clinicopathological data and the relative efficacy information. To assess inter-observer reproducibility, the intraclass correlation coefficient (ICC) was calculated for each radiomic feature. Only features demonstrating satisfactory inter-observer reproducibility, defined as an ICC of 0.80 or higher, were retained in the model.

For each radiomic model, the following feature selection process was carried out in four steps (1): Features with a variance greater than 1.0 were selected by variance threshold (2); The Mann-Whitney U test was applied to select features associated with NAC treatment relative efficacy (3); Feature importance was ranked using a random forest model, and the top 100 most important features were selected (4); Least absolute shrinkage and selection operator (LASSO) regression with 10-fold cross-validation was used to select features with non-zero coefficients.

### 2.7 Radiomic model construction and validation

First, A total of 9 basic radiomic models were constructed. These models were built using the intratumoral, peritumoral, and intratumoral + peritumoral image features of DCE images; the intratumoral, peritumoral, and intratumoral + peritumoral image features of ADC images; as well as the intratumoral, peritumoral, and intratumoral + peritumoral image features of both DCE and ADC images.

The radiomic models were developed using an extreme gradient boosting (XGBoost) algorithm, based on the selected radiomic features. A grid search method and five-fold cross-validation were conducted to determine the optimal hyperparameters for the model. Four folds (80% of the patients) were utilized for training the model, while the remaining fold (20% of the patients) was used to select the optimal hyperparameters. The hyperparameters ‘learning_rate’, ‘n_estimators’, and ‘max_depth’ were used in the grid search for model development. To ensure the robustness of the model, the entire construction process was replicated 1000 times using the bootstrap method. The effectiveness of the models was evaluated by analyzing their Receiver Operating Characteristic (ROC) curves in both training and test sets. An optimal model, named the Original model, was selected from the 9 models based on the AUC value.

For the construction of the Delta model, the differences in the features of Original model between the pre-NAC and mid-NAC stages were calculated. Specifically, for each feature, the value at the mid-NAC stage was subtracted from the value at the pre-NAC stage. These calculated differences served as the new feature set for the Delta model.

For the Fusion model, we employed a union of features from both the Original model and the Delta model. Specifically, the Fusion model incorporates both the feature values from the pre-NAC and mid-NAC stages themselves and the differences in feature values between these stages.

The XGBoost algorithm, which was the same as that used in the construction of the Original model, was employed to train the Delta model and the Fusion model.

### 2.8 Hybrid model construction and validation

To construct hybrid models, the outputs of the Original, Delta, and Fusion radiomic models were used as radiomic signatures. Important clinicopathological variables are separately selected for each radiomic signature in the training set. Specifically, individual logistic regression models were established for each radiomic signature, incorporating all clinicopathological variables. These models were used to evaluate the association of the combined variables with the relative efficacy in NAC. Backward stepwise selection based on the Akaike information criterion was then performed to identify important clinicopathological variables for each radiomic signature.

Hybrid models were constructed using logistic regression, which combined each radiomic signature with corresponding important clinicopathological variables. To select the optimal hyperparameters for the model, a grid search method combined with five-fold cross-validation was implemented. The hyperparameters ‘solver’, ‘penalty’, and ‘C’ were used in the grid search for model development. Additionally, for comparative purposes, a clinicopathological model that exclusively contained all clinicopathological variables was also established.

### 2.9 Statistical analysis

Chi-square tests were used to compare clinicopathological characteristics between patients in different groups or sets using categorical variables. Model performance was evaluated by the area under the receiver operating characteristic curve (AUC). Sensitivity, specificity, accuracy, positive predictive value (PPV), and negative predictive value (NPV) were calculated for both the training and test sets. The 95% confidence intervals (CI) for each metric were computed using the bootstrap method with 1000 intervals. The optimal cutoff value for the radiomic score in the training set was determined by maximizing the Youden index, and these fixed cutoff values were subsequently applied to the test set. All statistical tests were two-sided, with statistical significance indicated by a P value <0.05. All statistical analyses were performed using R 4.2.3 or Python 3.9.13.

## 3 Results

### 3.1 Baseline characteristics of patients

A total of 303 patients were excluded from the study due to insufficient MRI data (n=292) or inadequate image quality (n=11). Consequently, 190 patients were included in the study. **Table 2** summarizes the baseline characteristics of all patients. The proportion of patients for whom AC was superior to T was 48.1% (64 out of 133) in the training set and 47.4% (27 out of 57) in the test set. No significant differences in clinicopathological characteristics were observed between patients for whom AC was superior to T and those for whom T was superior to AC in either set (all p-values > 0.05).

**Table 2:** Characteristics of the patients in the training and the test sets.

Characteristics	Training Set (n=133)			Test Set (n=57)		
	AC superior to T (n=64)	T superior to AC (n=69)	P Value	AC superior to T (n=27)	T superior to AC (n=30)	P Value
Age			0.970			1.000
<50	29 (45)	30 (43)		17 (63)	18 (60)	
≥ 50	35 (55)	39 (57)		10 (37)	12 (40)	
Menstrual status			0.817			0.966
Premenopausal	33 (52)	38 (55)		16 (59)	19 (63)	
Postmenopausal	31 (48)	31 (45)		11 (41)	11 (37)	
clinical N stage			1.000			0.925
cN0	9 (14)	10 (14)		2 (7)	1 (3)	
cN1-3	55 (86)	59 (86)		25 (93)	29 (97)	
clinical T stage			0.203			1.000
cT1–2	46 (72)	57 (83)		21 (78)	23 (77)	
cT3–4 or cTx	18 (28)	12 (17)		6 (22)	7 (23)	
ER status			0.668			0.902
Positive	51 (80)	58 (84)		24 (89)	28 (93)	
Negative	13 (20)	11 (16)		3 (11)	2 (7)	
PR status			1.000			0.715
Positive	38 (59)	42 (61)		21 (78)	21 (70)	
Negative	26 (41)	27 (39)		6 (22)	9 (30)	
HER2 status			0.379			1.000
Positive	5 (8)	2 (3)		0 (0)	1 (3)	
Negative	59 (92)	67 (97)		27 (100)	29 (97)	
Ki67			0.508			0.112
Low proliferation (<30%)	17 (27)	23 (33)		7 (26)	15 (50)	
High proliferation (≥30%)	47 (73)	46 (67)		20 (74)	15 (50)	

Unless stated otherwise, the data represent the number of patients, with percentages in parentheses. AC, anthracycline and cyclophosphamide; T, taxanes; ER, estrogen receptor; PR, progesterone receptor; HER2, human epidermal growth factor receptor 2.

### 3.2 Development and performance of radiomic models

First, nine radiomic models were constructed by integrating features from specific MRI sequences and regions. Details of the selected features for the nine radiomic models are provided in **Supplementary Table S1**. **Table 3** provides the performance metrics of each radiomic model within the training and test sets. In the training set, the DCE+ADC-tumor+peri model yielded the best prediction with an AUC of 0.864 (95% CI: 0.792-0.911). In the test set, models utilizing the combined DCE+ADC sequences demonstrated relatively higher and more stable performance compared to models using either the DCE or ADC sequence alone. Specifically, the AUCs were 0.663 (95% CI: 0.549-0.767) for the DCE+ADC-tumor model, 0.598 (95% CI: 0.502-0.690) for the DCE+ADC-peri model, and 0.585 (95% CI: 0.480-0.677) for the DCE+ADC-tumor+peri model. Given the significance of peritumoral features, which reflect the tumor microenvironment and offer valuable insights into tumor behavior, the multi-sequence, multi-region DCE+ADC-tumor+peri model was consequently chosen as the Original model.

**Table 3:** Performance of radiomic models constructed by integrating features from specific MRI sequences and regions, within the training and test sets.

Datasets	Sequence	Region	AUC (95% CI)	ACC	SEN	SPE	PPV	NPV
Training Set	DCE	tumor	0.823 (0.735-0.879)	0.771	0.748	0.792	0.773	0.779
		peri	0.798 (0.730-0.854)	0.738	0.732	0.745	0.728	0.759
		tumor+peri	0.837 (0.760-0.885)	0.780	0.765	0.795	0.783	0.790
	ADC	tumor	0.813 (0.742-0.866)	0.757	0.777	0.737	0.738	0.789
		peri	0.804 (0.727-0.856)	0.748	0.708	0.785	0.760	0.752
		tumor+peri	0.830 (0.750-0.879)	0.772	0.759	0.785	0.772	0.784
	DCE+ADC	tumor	0.827 (0.753-0.876)	0.775	0.744	0.804	0.783	0.777
		peri	0.836 (0.763-0.887)	0.783	0.748	0.816	0.797	0.782
		tumor+peri	0.864 (0.792-0.911)	0.809	0.779	0.837	0.821	0.808
Test Set	DCE	tumor	0.481 (0.336-0.605)	0.489	0.410	0.560	0.449	0.514
		peri	0.583 (0.464-0.692)	0.548	0.450	0.636	0.524	0.565
		tumor+peri	0.544 (0.436-0.641)	0.524	0.419	0.618	0.499	0.542
	ADC	tumor	0.684 (0.550-0.785)	0.643	0.665	0.622	0.620	0.679
		peri	0.524 (0.429-0.634)	0.536	0.407	0.651	0.526	0.547
		tumor+peri	0.652 (0.543-0.749)	0.615	0.572	0.653	0.605	0.634
	DCE+ADC	tumor	0.663 (0.549-0.767)	0.625	0.508	0.730	0.638	0.625
		peri	0.598 (0.502-0.690)	0.557	0.454	0.649	0.545	0.570
		tumor+peri	0.585 (0.480-0.677)	0.564	0.445	0.671	0.550	0.575

DCE, dynamic contrast-enhanced; ADC, apparent diffusion coefficient; tumor, intratumoral regions; peri, peritumoral regions. AUC, area under the curve; CI, confidence interval; ACC, accuracy; SEN, sensitivity; SPE, specificity; PPV, positive predictive value; NPV, negative predictive value.

Subsequently, three radiomic models were developed, each based on different aspects of radiomic features: the Original model, the Delta model, and the Fusion model. Details of the selected features for the Original, Delta, and Fusion models are provided in **Supplementary Table S2**. **Table 4** summarizes the AUC, accuracy, sensitivity, specificity, PPV, and NPV for each radiomic model within the training and test sets. The Delta model outperformed the Original model with an AUC of 0.887 (95% CI: 0.816-0.930) in the training set and 0.757 (95% CI: 0.683-0.817) in the test set. The Fusion model did not show improved performance over the Delta model, with an AUC of 0.887 (95% CI: 0.822-0.931) in the training set and 0.749 (95% CI: 0.644-0.837) in the test set.

**Table 4:** Performance of radiomic models, hybrid models and clinicopath model within the training and test sets.

Datasets	Model	AUC (95% CI)	ACC	SEN	SPE	PPV	NPV
Training Set	Radiomic model						
	Original	0.863 (0.786-0.910)	0.809	0.779	0.836	0.820	0.807
	Delta	0.887 (0.816-0.930)	0.827	0.818	0.835	0.826	0.836
	Fusion	0.887 (0.822-0.931)	0.831	0.822	0.838	0.829	0.839
	Hybrid model						
	Original + clinicopath	0.880 (0.864-0.886)	0.814	0.826	0.802	0.796	0.833
	Delta + clinicopath	0.887 (0.873-0.892)	0.819	0.852	0.788	0.789	0.853
	Fusion + clinicopath	0.894 (0.884-0.898)	0.823	0.848	0.801	0.800	0.853
	Clinicopath model	0.548 (0.500-0.601)	0.555	0.269	0.821	0.426	0.557
Test Set	Radiomic model						
	Original	0.589 (0.495-0.682)	0.566	0.447	0.673	0.553	0.577
	Delta	0.757 (0.683-0.817)	0.695	0.588	0.791	0.724	0.685
	Fusion	0.749 (0.644-0.837)	0.677	0.526	0.813	0.726	0.660
	Hybrid model						
	Original + clinicopath	0.599 (0.578-0.615)	0.571	0.312	0.804	0.588	0.566
	Delta + clinicopath	0.772 (0.744-0.786)	0.691	0.835	0.561	0.632	0.793
	Fusion + clinicopath	0.749 (0.728-0.764)	0.656	0.478	0.817	0.710	0.636
	Clinicopath model	0.534 (0.455-0.634)	0.530	0.205	0.823	0.332	0.541

Clinicopath, clinicopathological. AUC, area under the curve; CI, confidence interval; ACC, accuracy; SEN, sensitivity; SPE, specificity; PPV, positive predictive value; NPV, negative predictive value.

### 3.3 Development and performance of hybrid models

Hybrid models were constructed by combining radiomic features with clinical indicators to predict the relative efficacy in NAC. **Table 4** provides a summary of the AUC, accuracy, sensitivity, specificity, PPV, and NPV for each hybrid model in both the training and test sets. After separately incorporating radiomic signatures and corresponding important clinicopathological features into three hybrid models, the performance of Delta+clinicopath model continued to outperform the Original+clinicopath and Fusion+clinicopath models, achieving an AUC of 0.887 (95% CI: 0.873-0.892) in the training set and 0.772 (95% CI: 0.744-0.786) in the test set.

During the development of hybrid models, alongside the Original radiomic signature, menstrual status, clinical T stage, and HER2 status were identified as independent predictors of the relative efficacy in NAC. For the Delta radiomic signature, clinical T stage was identified as an independent predictor of the relative efficacy in NAC (**Table 5**). Similarly, the clinical T stage also independently predicted the relative efficacy in NAC for the Fusion radiomic signature.

**Table 5:** Backward stepwise-selected variables in constructing the Delta+clinicopath model.

Variable	β Coefficient	Odds Ratio (95% CI)	P Value
Intercept	-2.34	0.10 (0.03-0.33)	<0.001
Clinical T stage			
cT1–2	-0.81	0.45 (0.15-1.37)	0.159
cT3–4 or cTx	Reference		
Delta radiomic signature	6.20	490.57 (76.29-3154.58)	<0.001

Clinicopath, clinicopathological; CI, confidence interval.

**Figure 4:**
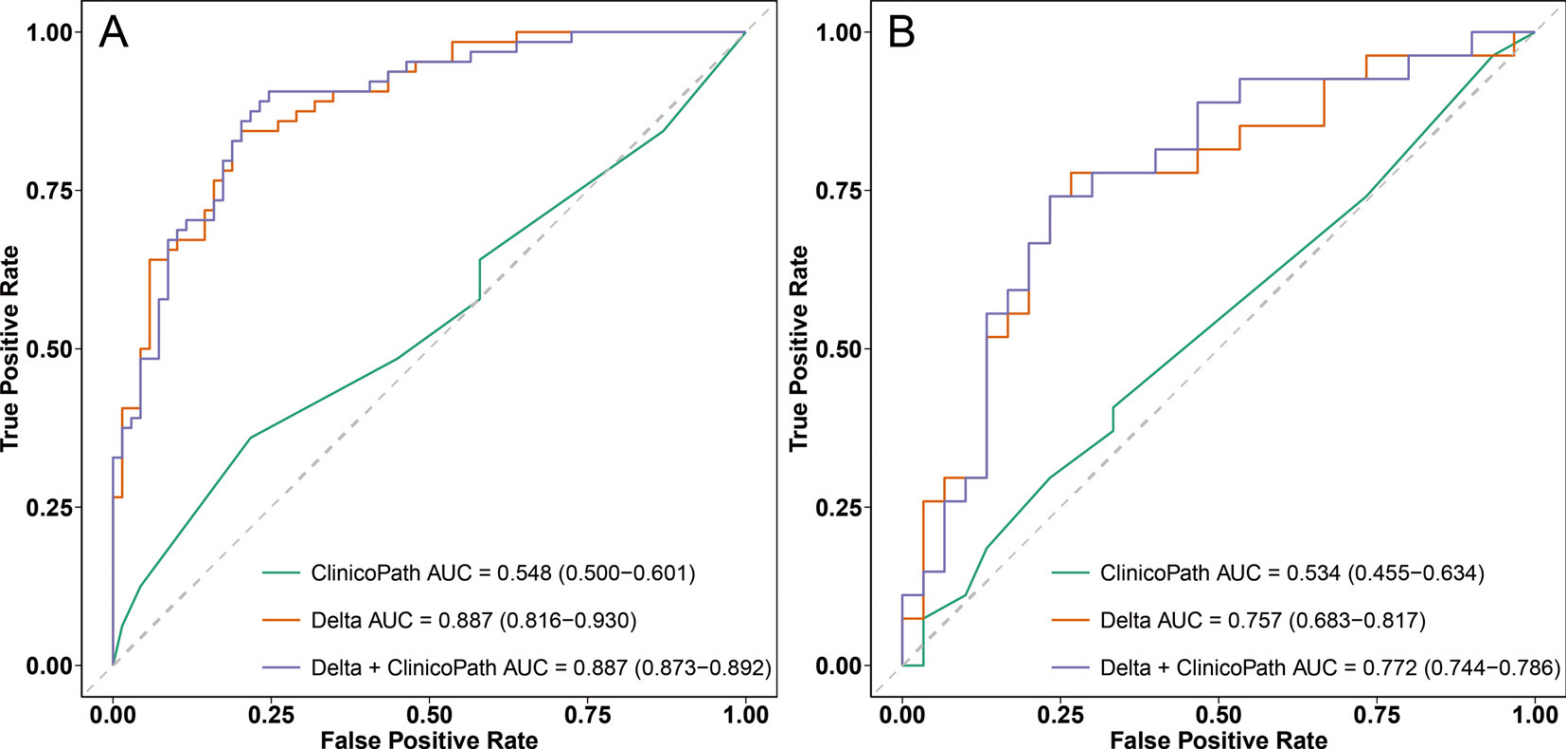
ROC curves of the clinicopath model, Delta model, and Delta+clinicopath model in the training set **(A)** and the test set **(B)**. ROC, receiver operating characteristic; AUC, area under the curve. Clinicopath, clinicopathological.

**Figure 4** displays the ROC curves for the most effective radiomic model (the Delta model), the most effective hybrid model (the Delta+clinicopath model) and the clinicopathological model alone. In the training set, both the Delta model and the Delta+clinicopath model achieved an AUC of 0.887. However, the Delta+clinicopath model exhibited a more concentrated performance, with a 95% CI ranging from 0.873 to 0.892, compared to the Delta model’s wider range of 0.816 to 0.930. In the test set, the Delta+clinicopath model produced a higher AUC of 0.772 in contrast to the Delta model’s 0.757. The combined performance on the training and test sets underscores the advantages of the Delta+clinicopath model in stratifying patients by predicting their relative efficacy in NAC.

**Figure 5:**
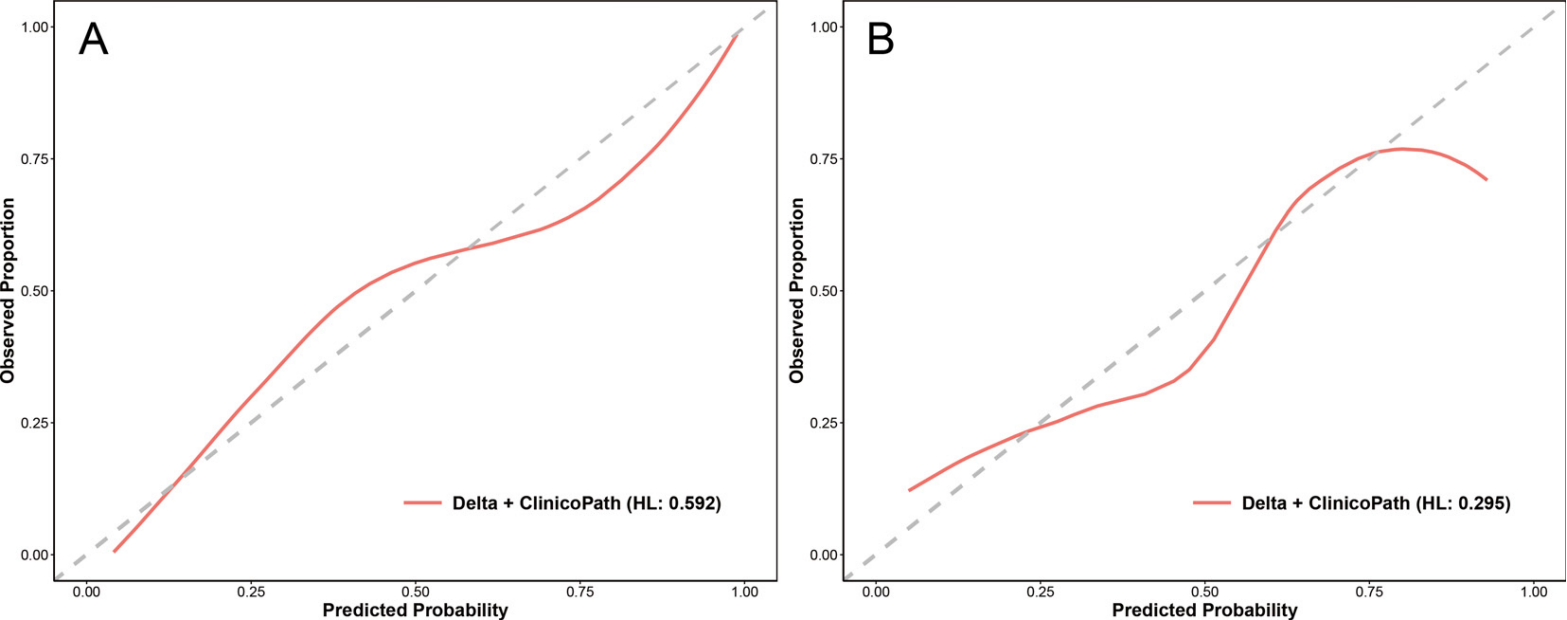
Calibration curves of the Delta+clinicopath model in the training set **(A)** and the test set **(B)**. Clinicopath, clinicopathological. HL, the p-value of the Hosmer-Lemeshow test.

**Figure 6:**
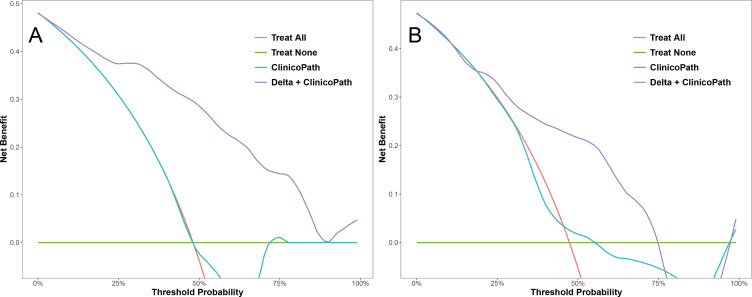
Decision curves of the clinicopath model and the Delta+clinicopath model in the training set **(A)** and the test set **(B)**. Clinicopath, clinicopathological.

For the optimal hybrid model, calibration curve analysis showed reasonable consistency between the predicted probabilities and actual outcomes regarding the relative efficacy in NAC in both the training and test sets (**Figure 5**). The Hosmer-Lemeshow test yielded non-significant results in both sets, with a p-value of 0.592 in the training set and 0.295 in the test set, indicating no significant deviation from a perfect model fit. DCA revealed that the optimal hybrid model delivered a substantial clinical net benefit at all threshold probabilities in the training set (as shown in **Figure 6A**) and between 0 and 0.74 in the test set (as shown in **Figure 6B**).

## 4 Discussion

Longitudinal monitoring of tumor response during NAC using imaging methods and adjusting subsequent regimens based on the treatment response is essential for personalized treatment of breast cancer and maximizing the potential of NAC (15, 16). Accurate prediction of the relative efficacy of regimens is crucial for optimizing NAC treatment plans. In the AC-T regimen, if the initial four cycles of AC demonstrate superior efficacy, extending the AC treatment may provide greater benefits to the patient. Conversely, if the initial four cycles of AC prove less effective, timely switching to T aligns with the patient’s best interest. However, few studies have explored how to predict the relative efficacy during the mid-term of NAC. In this study, we developed a multi-sequence, multi-region MRI radiomic model to predict the relative efficacy of four cycles of AC treatment followed by four cycles of T treatment, demonstrating robust performance. Additionally, integrating clinicopathological factors with radiomics significantly enhanced predictive accuracy, suggesting that our model could inform treatment adjustments during the mid-term of NAC.

The best model for predicting the relative efficacy of AC and T during NAC was the Delta + clinicopath model. A similar prospective trial by Guo et al. (17) demonstrated the predictive power of combining delta radiomic features with clinical indicators in predicting pathologic complete response after NAC. They developed a model combining delta radiomic features with clinical indicators, which achieved AUCs of 0.934 and 0.864 in the training and test sets, respectively, outperforming the model based solely on delta radiomic features. This result aligns with our study, highlighting the valuable contribution of breast cancer-related clinical or pathological factors in improving the accuracy of delta radiomics.

To capture the complex tumor microenvironment changes induced by NAC, we utilized a multi-sequence strategy with DCE and ADC. DCE is widely used in breast MRI radiomics studies for visualizing tumor vascularity, while ADC reflects tissue microstructure, cellular density, and membrane integrity (18). Prior studies have shown that combining DCE with ADC or DWI in radiomic models predicts NAC treatment response more accurately (19–22), and our results are consistent with these findings, underscoring the value of such combinations for evaluating NAC efficacy. Additionally, because peritumoral features such as lymphovascular invasion and angiogenesis are key prognostic factors (23, 24), and the efficacy of peritumoral radiomics has been preliminarily demonstrated in NAC response evaluation (25), we included both intratumoral and peritumoral regions in our multi-region strategy. For this multi-region strategy, our results are in line with previous studies that combine imaging features from multiple regions (26–29), showing improved predictive performance compared to using features from a single region. The role of multi-sequence and multi-region strategies can be observed by examining the features incorporated into the models. In the radiomic models (**Supplementary Table S1**), the DCE+ADC-tumor model included 8 DCE features and 6 ADC features, the DCE+ADC-peri model included 5 DCE features and 7 ADC features, and the DCE+ADC-tumor+peri model included 8 DCE features and 7 ADC features. This even distribution of DCE and ADC features across the models indicates the enhancement of predictive performance through multi-sequence combination. From a multi-region perspective, the DCE+ADC-tumor+peri model included 8 intratumoral and 7 peritumoral features, suggesting that the combination of multi-sequence and multi-region features captured richer tumor-related information, making it particularly effective for predicting relative efficacy in NAC.

However, this study has several limitations. First, while our model predicts relative efficacy between NAC regimens based on tumor volume changes, it is unclear whether adjusting treatment based on this prediction will improve pathological complete response or overall outcomes. This requires further validation through prospective trials. Second, although this study utilized the second post-contrast phase images of the DCE sequence for segmentation to improve reproducibility, it should be acknowledged that volumetric assessment of non-concentric regression tumors poses unique challenges, with MRI potentially overestimating or underestimating tumor volume in such cases. Third, the study’s retrospective design, small sample size, and single-center data limit the generalizability of the findings. Larger, multicenter studies are needed for broader validation. Fourth, the focus on the AC-T regimen may introduce biases, including the underrepresentation of HER2 positive cases, which will be addressed in future studies.

## 5 Conclusions

In this study, we developed a hybrid radiomic model that integrates clinical and biopsy pathology data with pre-NAC and mid-NAC breast MRI to predict the relative efficacy of AC and T treatments. This model demonstrated strong predictive performance and could serve as a valuable tool for guiding treatment decisions during NAC. By enabling early prediction of treatment response, the model holds potential for patient stratification and personalized adjustment of NAC regimens.

## Data Availability

The original contributions presented in the study are included in the article/- **Supplementary Material**. Further inquiries can be directed to the corresponding authors.

## Supplementary material

Supplementary Table 1 Details of selected features in 9 basic radiomic models.

Supplementary Table 2 Details of selected features in the Original model, the Delta model, and the Fusion model.
