# Supplementary material for: Predicting relative efficacy of anthracyclines and taxanes in breast cancer neoadjuvant AC-T chemotherapy using longitudinal MRI radiomic model
Source: Front Oncol. 2025 May 15;15:1544833. doi: 10.3389/fonc.2025.1544833 (PMC12119262; doi:10.3389/fonc.2025.1544833)
Supplement: Supplementary Table 1 — Details of selected features in 9 basic radiomic models. [file Table1.docx]

SUPPLEMENTARY TABLE 1 Details of selected features in 9 basic radiomic models.

| **Features for DCE-tumor model** | |
| --- | --- |
| wavelet-LHH_firstorder_Mean_A | original_glcm_Correlation_delta |
| wavelet-HLL_gldm_LargeDependenceHighGrayLevelEmphasis_A | log-sigma-2-0-mm-3D_glszm_HighGrayLevelZoneEmphasis_delta |
| wavelet-HLH_glrlm_LongRunLowGrayLevelEmphasis_A | log-sigma-4-0-mm-3D_glcm_Idn_delta |
| log-sigma-1-0-mm-3D_firstorder_90Percentile_B | log-sigma-5-0-mm-3D_glcm_Imc1_delta |
| log-sigma-4-0-mm-3D_gldm_LargeDependenceHighGrayLevelEmphasis_B | wavelet-LHH_glszm_SmallAreaLowGrayLevelEmphasis_delta |
| log-sigma-5-0-mm-3D_glrlm_GrayLevelNonUniformity_B |  |
| **Features for DCE-peri model** | |
| wavelet-LHL_glszm_LargeAreaEmphasis_A | wavelet-HLH_glszm_SizeZoneNonUniformity_B |
| original_firstorder_10Percentile_B | wavelet-HHH_glcm_ClusterTendency_B |
| log-sigma-2-0-mm-3D_glcm_ClusterProminence_B | log-sigma-2-0-mm-3D_firstorder_Skewness_delta |
| log-sigma-2-0-mm-3D_glszm_LargeAreaLowGrayLevelEmphasis_B | log-sigma-3-0-mm-3D_glcm_Imc2_delta |
| log-sigma-5-0-mm-3D_firstorder_90Percentile_B | wavelet-LLH_glszm_LargeAreaHighGrayLevelEmphasis_delta |
| wavelet-LHH_gldm_LargeDependenceLowGrayLevelEmphasis_B | wavelet-HLL_firstorder_Median_delta |
| wavelet-HLL_glcm_ClusterShade_B | wavelet-HLL_glcm_Imc2_delta |
| wavelet-HLL_glszm_LargeAreaLowGrayLevelEmphasis_B | wavelet-LLL_firstorder_10Percentile_delta |
| **Features for DCE-tumor+peri model** | |
| wavelet-LLH_gldm_LargeDependenceLowGrayLevelEmphasis_A (tumor) | log-sigma-2-0-mm-3D_firstorder_Skewness_delta (peri) |
| log-sigma-2-0-mm-3D_glcm_ClusterProminence_B (peri) | log-sigma-3-0-mm-3D_glcm_Imc2_delta (peri) |
| log-sigma-4-0-mm-3D_firstorder_Variance_B (tumor) | log-sigma-4-0-mm-3D_glcm_Idn_delta (tumor) |
| wavelet-HLL_glcm_ClusterShade_B (peri) | log-sigma-5-0-mm-3D_gldm_DependenceEntropy_delta (tumor) |
| wavelet-LLL_firstorder_10Percentile_B (peri) | wavelet-HHL_glcm_Imc2_delta (peri) |
| **Features for ADC-tumor model** | |
| wavelet-HHL_firstorder_Skewness_A | wavelet-LLH_gldm_LargeDependenceHighGrayLevelEmphasis_delta |
| wavelet-HHL_glcm_ClusterShade_A | wavelet-LHL_glrlm_GrayLevelNonUniformity_delta |
| log-sigma-5-0-mm-3D_glszm_LargeAreaHighGrayLevelEmphasis_B | wavelet-LHH_glrlm_LongRunEmphasis_delta |
| wavelet-LHH_glszm_SmallAreaLowGrayLevelEmphasis_B | wavelet-LHH_glrlm_LongRunHighGrayLevelEmphasis_delta |
| wavelet-HLL_gldm_LargeDependenceLowGrayLevelEmphasis_B | wavelet-LHH_glrlm_ShortRunLowGrayLevelEmphasis_delta |
| original_glcm_Imc2_delta | wavelet-HLH_glcm_Imc2_delta |
| log-sigma-3-0-mm-3D_glcm_Imc1_delta | wavelet-HHL_firstorder_Range_delta |
| log-sigma-5-0-mm-3D_glrlm_LongRunHighGrayLevelEmphasis_delta | wavelet-HHL_glrlm_RunVariance_delta |
| **Features for ADC-peri model** | |
| original_firstorder_10Percentile_B | log-sigma-4-0-mm-3D_firstorder_Skewness_delta |
| log-sigma-3-0-mm-3D_glrlm_ShortRunLowGrayLevelEmphasis_B | log-sigma-5-0-mm-3D_firstorder_Skewness_delta |
| log-sigma-2-0-mm-3D_glrlm_LongRunLowGrayLevelEmphasis_delta | wavelet-LLH_gldm_SmallDependenceLowGrayLevelEmphasis_delta |
| log-sigma-3-0-mm-3D_glcm_Idn_delta | wavelet-HLL_glcm_Imc2_delta |
| **Features for ADC-tumor+peri model** | |
| log-sigma-3-0-mm-3D_glrlm_ShortRunLowGrayLevelEmphasis_B (peri) | log-sigma-3-0-mm-3D_glcm_Idn_delta (peri) |
| wavelet-LHH_glszm_SmallAreaLowGrayLevelEmphasis_B (tumor) | wavelet-LLH_gldm_SmallDependenceLowGrayLevelEmphasis_delta (peri) |
| wavelet-HHL_glszm_HighGrayLevelZoneEmphasis_B (tumor) | wavelet-LHH_glrlm_LongRunHighGrayLevelEmphasis_delta (tumor) |
| log-sigma-3-0-mm-3D_firstorder_Skewness_delta (peri) | wavelet-LHH_glrlm_RunVariance_delta (tumor) |
| **Features for DCE+ADC-tumor model** | |
| wavelet-LLH_glcm_Autocorrelation_A (DCE) | log-sigma-4-0-mm-3D_glszm_ZonePercentage_delta (DCE) |
| wavelet-HLH_glrlm_LowGrayLevelRunEmphasis_A (DCE) | wavelet-HHL_firstorder_Skewness_A (ADC) |
| log-sigma-3-0-mm-3D_firstorder_Maximum_B (DCE) | log-sigma-4-0-mm-3D_glcm_ClusterShade_B (ADC) |
| log-sigma-5-0-mm-3D_glrlm_GrayLevelVariance_B (DCE) | wavelet-LLH_glszm_LargeAreaHighGrayLevelEmphasis_B (ADC) |
| log-sigma-2-0-mm-3D_glszm_HighGrayLevelZoneEmphasis_delta (DCE) | original_gldm_LargeDependenceLowGrayLevelEmphasis_delta (ADC) |
| log-sigma-3-0-mm-3D_glcm_JointEntropy_delta (DCE) | wavelet-HLH_glrlm_ShortRunEmphasis_delta (ADC) |
| log-sigma-4-0-mm-3D_glcm_Idn_delta (DCE) | wavelet-HHL_firstorder_Range_delta (ADC) |
| **Features for DCE+ADC-peri model** | |
| wavelet-LHL_glcm_DifferenceVariance_B (DCE) | log-sigma-3-0-mm-3D_glrlm_ShortRunLowGrayLevelEmphasis_B (ADC) |
| wavelet-LLL_firstorder_10Percentile_B (DCE) | log-sigma-3-0-mm-3D_firstorder_Skewness_delta (ADC) |
| log-sigma-2-0-mm-3D_firstorder_Skewness_delta (DCE) | log-sigma-3-0-mm-3D_glcm_Idn_delta (ADC) |
| log-sigma-3-0-mm-3D_glcm_Imc2_delta (DCE) | log-sigma-4-0-mm-3D_firstorder_Skewness_delta (ADC) |
| wavelet-HLL_firstorder_Median_delta (DCE) | log-sigma-5-0-mm-3D_glrlm_ShortRunHighGrayLevelEmphasis_delta (ADC) |
| wavelet-LHH_glrlm_RunLengthNonUniformity_A (ADC) | wavelet-LLH_gldm_SmallDependenceLowGrayLevelEmphasis_delta (ADC) |
| **Features for DCE+ADC-tumor+peri model** | |
| wavelet-LHL_glszm_LargeAreaEmphasis_A (DCE-peri) | wavelet-HHL_firstorder_Skewness_A (ADC-tumor) |
| wavelet-HLH_glcm_Autocorrelation_A (DCE-tumor) | log-sigma-2-0-mm-3D_glcm_Imc2_delta (ADC-tumor) |
| original_firstorder_10Percentile_B (DCE-peri) | log-sigma-3-0-mm-3D_glcm_Idn_delta (ADC-peri) |
| log-sigma-4-0-mm-3D_glrlm_GrayLevelVariance_B (DCE-tumor) | log-sigma-3-0-mm-3D_gldm_SmallDependenceLowGrayLevelEmphasis_delta (ADC-peri) |
| wavelet-LHH_gldm_LargeDependenceLowGrayLevelEmphasis_B (DCE-peri) | log-sigma-4-0-mm-3D_firstorder_Skewness_delta (ADC-peri) |
| log-sigma-4-0-mm-3D_glcm_Idn_delta (DCE-tumor) | wavelet-LHH_glrlm_LongRunHighGrayLevelEmphasis_delta (ADC-tumor) |
| log-sigma-4-0-mm-3D_glszm_LowGrayLevelZoneEmphasis_delta (DCE-peri) | wavelet-HLH_glcm_Imc2_delta (ADC-tumor) |
| wavelet-LHH_glrlm_ShortRunEmphasis_delta (DCE-tumor) |  |

DCE, dynamic contrast-enhanced; ADC, apparent diffusion coefficient. Tumor, intratumoral regions; peri, peritumoral regions. A, pre-NAC; B, mid-NAC; delta, the difference in the feature between pre-NAC and mid-NAC stages. NAC, neoadjuvant chemotherapy. GLCM, gray-level co-occurrence matrix; GLDM, gray-level dependence matrix; GLRLM, gray-level run length matrix; GLSZM, gray-level size zone matrix.
