# Supplementary material for: Predicting relative efficacy of anthracyclines and taxanes in breast cancer neoadjuvant AC-T chemotherapy using longitudinal MRI radiomic model
Source: Front Oncol. 2025 May 15;15:1544833. doi: 10.3389/fonc.2025.1544833 (PMC12119262; doi:10.3389/fonc.2025.1544833)
Supplement: Supplementary Table 2 — Details of selected features in the Original model, the Delta model, and the Fusion model. [file Table2.docx]

SUPPLEMENTARY TABLE 2 Details of selected features in the Original model, the Delta model, and the Fusion model.

| **Features for the Original model** |  |
| --- | --- |
| wavelet-LHL_glszm_ZoneVariance_A (DCE-peri) | log-sigma-5-0-mm-3D_glrlm_GrayLevelVariance_B (DCE-tumor) |
| wavelet-LHH_firstorder_Skewness_A (DCE-peri) | wavelet-LHL_glszm_SmallAreaLowGrayLevelEmphasis_B (DCE-peri) |
| wavelet-HLL_glrlm_ShortRunHighGrayLevelEmphasis_A (DCE-tumor) | wavelet-LHH_glrlm_ShortRunHighGrayLevelEmphasis_B (DCE-peri) |
| original_firstorder_10Percentile_B (DCE-peri) | wavelet-HLL_glcm_ClusterShade_B (DCE-peri) |
| log-sigma-3-0-mm-3D_gldm_LargeDependenceLowGrayLevelEmphasis_B (DCE-tumor) | log-sigma-2-0-mm-3D_glszm_LargeAreaHighGrayLevelEmphasis_B (ADC-peri) |
| log-sigma-4-0-mm-3D_gldm_LargeDependenceLowGrayLevelEmphasis_B (DCE-peri) | log-sigma-3-0-mm-3D_gldm_LowGrayLevelEmphasis_B (ADC-peri) |
| **Features for the Delta model** |  |
| log-sigma-2-0-mm-3D_firstorder_Skewness_delta (DCE-peri) | log-sigma-4-0-mm-3D_firstorder_Skewness_delta (ADC-peri) |
| log-sigma-3-0-mm-3D_glcm_Imc2_delta (DCE-peri) | log-sigma-5-0-mm-3D_glcm_ClusterShade_delta (ADC-peri) |
| log-sigma-4-0-mm-3D_gldm_LargeDependenceEmphasis_delta (DCE-tumor) | log-sigma-5-0-mm-3D_glszm_GrayLevelVariance_delta (ADC-peri) |
| wavelet-LLH_glrlm_LowGrayLevelRunEmphasis_delta (DCE-peri) | wavelet-LLH_gldm_LargeDependenceHighGrayLevelEmphasis_delta (ADC-tumor) |
| wavelet-LHH_glrlm_ShortRunEmphasis_delta (DCE-tumor) | wavelet-LLH_gldm_SmallDependenceLowGrayLevelEmphasis_delta (ADC-peri) |
| wavelet-HLL_firstorder_Median_delta (DCE-peri) | wavelet-HLH_glcm_Imc2_delta (ADC-tumor) |
| log-sigma-2-0-mm-3D_glrlm_LongRunLowGrayLevelEmphasis_delta (ADC-peri) | wavelet-HHL_firstorder_Range_delta (ADC-tumor) |
| log-sigma-3-0-mm-3D_glcm_Idn_delta (ADC-peri) |  |
| **Features for the Fusion model** | |
| wavelet-LHL_glszm_LargeAreaEmphasis_A (DCE-peri) | wavelet-HHL_firstorder_Skewness_A (ADC-tumor) |
| wavelet-HLH_glcm_Autocorrelation_A (DCE-tumor) | log-sigma-2-0-mm-3D_glcm_Imc2_delta (ADC-tumor) |
| original_firstorder_10Percentile_B (DCE-peri) | log-sigma-3-0-mm-3D_glcm_Idn_delta (ADC-peri) |
| log-sigma-4-0-mm-3D_glrlm_GrayLevelVariance_B (DCE-tumor) | log-sigma-3-0-mm-3D_gldm_SmallDependenceLowGrayLevelEmphasis_delta (ADC-peri) |
| wavelet-LHH_gldm_LargeDependenceLowGrayLevelEmphasis_B (DCE-peri) | log-sigma-4-0-mm-3D_firstorder_Skewness_delta (ADC-peri) |
| log-sigma-4-0-mm-3D_glcm_Idn_delta (DCE-tumor) | wavelet-LHH_glrlm_LongRunHighGrayLevelEmphasis_delta (ADC-tumor) |
| log-sigma-4-0-mm-3D_glszm_LowGrayLevelZoneEmphasis_delta (DCE-peri) | wavelet-HLH_glcm_Imc2_delta (ADC-tumor) |
| wavelet-LHH_glrlm_ShortRunEmphasis_delta (DCE-tumor) |  |

DCE, dynamic contrast-enhanced; ADC, apparent diffusion coefficient. Tumor, intratumoral regions; peri, peritumoral regions. A, pre-NAC; B, mid-NAC; delta, the difference in the feature between pre-NAC and mid-NAC stages. NAC, neoadjuvant chemotherapy. GLCM, gray-level co-occurrence matrix; GLDM, gray-level dependence matrix; GLRLM, gray-level run length matrix; GLSZM, gray-level size zone matrix.
